# Treatment of status epilepticus in pediatrics: curriculum learning combined with in-situ simulations

**DOI:** 10.1186/s12909-022-03626-x

**Published:** 2022-07-19

**Authors:** Huiping Wei, Hui Zhao, Ziming Huang, Xinyun Lei, Ming He, Ran Dong, Jiannan Wu, Jing Yue

**Affiliations:** Department of Emergence, Maternal and Child Health Hospital of Hubei Province, Tongji Medical College, Huazhong University of Science and Technology, Wuhan, 430070 China

**Keywords:** Status epilepticus, Pediatric intensive care unit, Emergency department, Curriculum learning, In situ simulation, Teamwork, Children

## Abstract

**Background:**

Appropriate and timely treatment of status epilepticus (SE) reduces morbidity and mortality. Therefore, skill-based identification and management are critical for emergency physicians.

**Purpose:**

To assess whether the ability of training physicians, residents, nurses, and others to respond to SE as a team could be improved by using curriculum learning [Strategies and Tools to Enhance Performance and Patient Safety of Team (TeamSTEPPS) course training] combined with in-situ simulations of emergency department (ED) staff.

**Approach:**

A pre-training-post-training design was used on SE skills and teamwork skills. Emergency training, residents, and N1 and N2 nurses completed the SE skill and teamwork assessments (pre-training) through in-situ simulation. Next, the participating physicians and nurses attended the SE course [Strategies and Tools to Enhance Performance and Patient Safety of Team (TeamSTEPPS) course training], followed by conscious skill practice, including in-situ simulation drills every 20 days (eight times total) and deliberate practice in the simulator. The participants completed the SE skill and teamwork assessments (post-training) again in an in-situ simulation. Pre-training-post-training simulated SE skills and teamwork performance were assessed. The simulation training evaluation showed that the training process was reasonable, and the training medical staff had different degrees of benefit in increasing subject interest, improving operational skills, theoretical knowledge, and work self-confidence.

**Findings:**

Sixty doctors and nurses participated in the intervention. When comparing the SE skills of 10 regular training physicians pre-training and post-training, their performance improved from 40% (interquartile range (IQR): 0–1) before training to 100% (IQR: 80.00–100) after training (*p* < 0.001). The teamwork ability of the 10 teams improved from 2.43 ± 0.09 before training to 3.16 ± 0.08 after training *(p* < 0.001).

**Conclusion:**

SE curriculum learning combined with in-situ simulation training provides the learners with SE identification and management knowledge in children and teamwork skills.

**Supplementary Information:**

The online version contains supplementary material available at 10.1186/s12909-022-03626-x.

## Introduction

Status epilepticus (SE) is a fixed convulsion state formed by continuous and frequent convulsions. Its traditional definition includes a convulsion lasting more than 5, 10, or 15 min (depending upon the guidelines) or continuous attacks, and the consciousness does not fully recover to baseline during intermissions. SE is the most common acute and severe neurological disease in pediatrics. Convulsion lasting more than 5–10 min is difficult to relieve by itself without appropriate anticonvulsant treatment. Thus, many authors tend to shorten the definition of convulsion duration (or “operational definition”) to 5 min to emphasize the importance of early treatment [1]. A course exceeding 1 h can result in sequelae [2]. In Western countries, the incidence of SE is 135–156 per 100,000 infants < 1 year old and 10–58 per 100,000 children aged 1–19 years [3], and the mortality is < 3% [4]. SE is the cause of death for about 25% of the children admitted to the pediatric intensive care unit (PICU) [5]. The incidence of SE in children in China is 18–20 per 100,000 children, and the mortality is 2%-7% [6, 7]. Therefore, improving the emergency department medical staff’s ability to treat SE and properly and quickly terminate the clinical attack will help reduce the disability and mortality rates and improve the prognosis [8, 9].

Traditionally, emergency residents learn about the diagnosis and management of SE by reading and listening to lectures, but experiential learning does not guarantee that medical students are proficient in the skills they are expected to be able to practice independently [10–13]. Simulation training is an educational discipline that uses simulation technology to create highly simulated patient and clinical scenarios and is recognized as an effective way to improve healthcare, reduce medical errors, and improve physician training, competency maintenance, medical process, theoretical basis, and practical experience [14]. In-situ simulation helps build a good team by training in basic and advanced life support first aid operations using virtual emergency scenarios, procedural simulations, CPR models, standardized patient communication scenarios, high-end simulators, virtual reality games, and virtual reality first aid simulators, to assure a management model that improves medical quality and patient safety [15].

Proficient skills in identifying and managing SE require quick thinking and planning, knowledge about the diagnostic process and treatment options, and teamwork and effective communication skills with other healthcare professionals such as nurses [16], but SE requires special milestones, as previously described [17, 18]. Simulation-based learning allows clinicians to develop these skills in a safe and controlled environment and ensures that they are prepared for independent patient practice. In addition, several studies have shown that curriculum learning combined with simulation training achieves better learning outcomes than simulation alone and traditional experiential learning [10, 11, 13, 19–21].

At present, training for pediatric SE mainly involves infants and newborns with convulsions due to fever, sepsis, electrolyte imbalance, drug intake, and pyridoxine-dependent convulsion [22–25]. Moreover, the limitations reported in some publications include a small sample size and only doctors without considering the doctor-nurse interactions [26].

Therefore, to explore the SE training model for pediatric emergency residents, a training method of curriculum learning combined with in-situ simulation was selected to create realistic patient encounters that require learners to take action in emergencies. This study was a quasi-experimental single-group pre-training/post-training study. It can be hypothesized that a combination of curriculum learning [Strategies and Tools to Enhance Performance and Patient Safety of Team (TeamSTEPPS) courses] and on-site simulation training could improve and enhance SE identification, management, and first aid skills among emergency team members.

## Subjects and methods

### Settings and participants

This quasi-experiment study was conducted from May 18 to December 31, 2020, at the Maternity and Child Health Hospital in Hubei Province. Our facility is a 1400-bed teaching hospital with approximately 170,000 annual pediatric visits to the emergency department. The education, work experience, and number of participants are shown in Supplementary Table S1. All participants who could not participate in the entire study training and evaluations due to physical illness or work obligations were excluded.

This work has been carried out in accordance with the Declaration of Helsinki (2000) of the World Medical Association. This study was approved by the Ethic Committee of omen And Children’s Hospital of Hubei Province (2021LW028), and all participants provided written informed consent.

### Study design

**Fig. 1:**
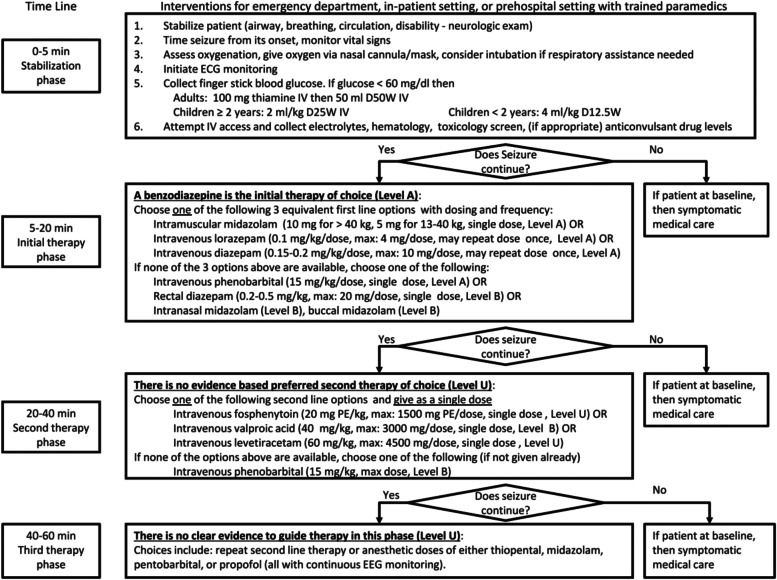
Proposed treatment algorithm for status epilepticus (20)

According to the emergency procedures of SE (Fig. 1) [27], in situ simulation scenarios and five different scripts for childhood SE (Table 1) were developed by two neurologists and two emergency medicine specialists. According to the purpose of the training, time deduction, and connection with the medical process, the emergency rescue of pediatric SE was divided into seven specific stages (Supplementary Table S2) [28, 29]: 1) stage 1: pre-inspection triage; 2) stages 2–6: in the rescue room; 3) stage 7: after successful cardiopulmonary resuscitation (ROSC), transfer to PICU and preparation of transfer materials and arrangement of transfer personnel.

**Table 1:** The cases used in the simulation

Case	Stages
1	A previously healthy 1-year-old male, fever for 5 days, manifested by altered mental status and seizures secondary to CNS infection
2	A 3-month-old female with intermittent seizures for 20 days with epileptic seizures with inherited metabolic disease
3	A 7-year-old male with sudden seizures for 10 min turned into an epileptic seizure
4	A previously healthy 4-day-old female presented with altered mental status and seizures secondary to pyridoxine deficiency
5	A previously healthy 2-year-old boy suffered a 15-min seizure after taking an organophosphorus pesticide by mistake

The Simbaby (1 to 3 years old)/SimJunior (5 to 9 years old) simulator (Nodo Medical, Norway) was used in the resuscitation room (in situ simulation). Each scenario was performed in the resuscitation room to ensure a high-fidelity environment.

The medical staff was divided into 10 groups, with six people in each group, including four nurses (N2 nurses served as the nurse team leader, with three N1 nurses) and two doctors (one standardized training physician and one resident physician) (Supplementary Table S1). The same individual played the same role in each scenario to maintain standardization and minimize bias. Each group completed an initial SE skill assessment and teamwork assessment (pre-training). Then, the participating doctors took the SE and TeamSTEPPS courses, and the participating nurses took the TeamSTEPPS courses (each course took 1 day). Conscious skill practice followed, including in-situ simulations every 20 days (a total of eight times) and deliberate practice in the simulator. The participants then completed the SE skill assessment and teamwork assessment again through the in-situ simulation (post-training). We compared the simulated SE skills and teamwork performance before and after training. The scripts for each set of in-situ simulations were randomly selected while ensuring that each script was executed twice per set. Ten in-situ simulation training results were recorded. The first result was taken as the pre-training and the last one as the post-training to compare the differences. Through the simulation training evaluation questionnaire, the participants could evaluate whether the training process was reasonable, whether it improved subject interest, whether it improved operational skills and theoretical knowledge, and whether it improved work self-awareness.

### Simulation training evaluation

A 26-item checklist was used to evaluate the mastery of SE management by the training physicians, and interrater reliability was very high (average κ = 0.99) across all items and judges [18]. The teamwork checklist was used to evaluate teamwork ability, which was found to have a high total content validity index of 0.96 [30]. In order to assess the inter-rater reliability on the checklist, four raters (two APLS-course directors and two pediatric epilepsy specialists) independently scored an identical random sample of 25% of pre-training and post-training videos. The raters were blinded to the status of the participants before or after the training. A panel of eight board-certified physicians with expertise in SE (two pediatric epilepsy specialists, three pediatric critical care medicine specialists, and three emergency medicine specialists) jointly confirmed the consistency and reliability of the scoring criteria. Surveys on curriculum satisfaction and self-confidence were scored on a Likert scale (1–5; 1 = strongly disagree, 5 = strongly agree).

### Statistical analysis

SPSS 16 (IBM, USA) was used for data analysis. Cohen’s kappa (κ) for interrater and test–retest reliability was calculated. Continuous data were presented as means ± standard deviation (mean ± SD). The paired t-test was used to compare the scores before and after training. Categorical data were presented as n (%). Fisher’s exact tests were used to evaluate differences between individual checklist items, comparing pre-training with post-training reassessment. *P*-values < 0.05 were considered statistically significant.

## Results

Sixty participants were enrolled in this study; none withdrew. There were 10 standardized training doctors (first-year postgraduate and first-year standardized training emergency physician) and 10 emergency resident doctors (graduated from postgraduate, emergency work less than 1 year). There were 40 nurses: 10 N2 nurses (bachelor’s degree, emergency work more than 1 year but less than 3 years; they completed the emergency specialist nurse training) and 30 N1 nurses (bachelor’s degree, emergency work less than or equal to 1 year; did not complete the emergency specialist nurse training). There were no differences in age, education, and working years among the teams. Prior to their participation, no participants knew the SE in-situ simulation training content. Supplementary Table S3 shows how many scenarios each group participated in.

All 20 physicians completed the first in-situ simulation assessment (pre-training), course training, and the final in-situ simulation assessment (post-training). Checklists were used to evaluate the participants’ management of the SE process (for the regular training physicians) and their teamwork ability (for the residents and groups) from the video recording. The interrater checklist reliability was very high (average *κ* = 0.86) across all items and judges.

Table 2 shows the checklist and the percentage of participants who completed each step correctly before and after the training. When focusing on specific key steps in SE management, only 3/10 of the regular training physicians correctly assessed the need for oral suction before but 10/10 correctly after the training (*p* < 0.001). Only 2/10 of the regular training physicians were performing a brief neurological exam before the training, and 9/10 were performing it after the training (*p* < 0.001). Only 2/10 of the regular training physicians were administering a second ASD prior to the training, while 9/10 were successfully administering an appropriate dose of a second ASD after the training (*p* < 0.001). Only 3/10 of the regular training physicians could correctly identify non-convulsive SE (NCSE) before the training, while all 10 regular training physicians could make the correct diagnosis after the training (*p* < 0.001).

**Table 2:** Skill items before and after the test, n (%)

Skill	Pre-training	Post-training	P (pre-post)
Evaluating the patient (before the patient has witnessed a seizure)			
Obtains a concise history (from RN/ED team/family members) (must ask all three to get credit)	2 (20%)	9 (90%)	< 0.001
States out loud the patient's level of alertness	2 (20%)	10 (100%)	< 0.001
Examines eyes	5 (50%)	9 (90%)	0.02
Examine for any focal weakness	6 (60%)	10 (100%)	0.02
Evaluating and stabilizing the patient (after witnessed seizure noted)			
States that the patient is having a seizure	5 (50%)	10 (100%)	0.01
States out loud time of seizure onset	1 (10%)	10 (100%)	< 0.001
Calls for help	8 (80%)	10 (100%)	0.53
Reposition patient on to the side	4 (40%)	9 (90%)	0.01
Evaluates patient's airway: suction patient	3 (30%)	10 (100%)	< 0.001
Places pulse oximeter	9 (90%)	10 (100%)	0.62
Asks RN to provide oxygen if the patient is hypoxic	8 (80%)	9 (90%)	0.58
Asks RN to check blood pressure	9 (90%)	10 (100%)	0.62
Asks RN to initiate telemonitoring (EKG) and ensures that this gets done by RN	6 (60%)	10 (100%)	0.02
Performs a brief neurological exam: must check or ask RN if any eye deviation	2 (20%)	10 (100%)	< 0.001
Management			
Ensures that the patient has a working IV access	4 (40%)	10 (100%)	0.01
Orders 1st-line ASD (must be given within 5 min of seizure onset)	10 (100%)	10 (100%)	> 0.999
Asks RN to check finger stick blood glucose	5 (50%)	10 (100%)	0.01
Orders labs	9 (90%)	100 (100%)	> 0.999
Order a second ASD (needs to be ordered within 5 min from ordering first ASD)	2 (20%)	9 (90%)	< 0.001
Calls pharmacy or asks RN to call the pharmacy to communicate the emergent need for ASDs	3 (30%)	10 (100%)	< 0.001
Orders a stat head CT	5 (50%)	10 (100%)	0.01
Communicates with attending physician/fellow on-call to staff the case	3 (30%)	10 (100%)	< 0.001
States out loud the concern for nonconvulsive status	3 (30%)	10 (100%)	< 0.001
Re-evaluating the case			
Orders a postload ASD level	0	8 (80%)	< 0.001
Orders an emergent EEG and call the on-call fellow to ask for a stat EEG	3 (30%)	10 (100%)	< 0.001
Makes appropriate decision regarding disposition/level of care (ICU) and communicates this decision to the nurse	4 (40%)	10 (100%)	0.01

The drug selection in this checklist was based on published guidelines at the time and prior to recent publications showing levetiracetam as an option for the treatment of status epilepticus [20]

*ASD* Antiseizure drug, *CBC* Complete blood count, *CMP* Complete metabolic profile, *CT* Computed tomography, *ED* Emergency department, *EEG* Electroencephalogram, *EKG* Electrocardiogram, *ICU* Intensive care unit, *IM* Intramuscular, *IV* Intravenous, *RN* Register nurse

TEAM scores were clustered at the mid-range (Table 3). Comparing the average scores of the 10 items before and after training, the average scores of all 10 items after training were significantly different from those before training (*p* < 0.05).

**Table 3:** TEAM scores before and after the test, mean ± SD

Item	Pre-training	Post-training	P (pre-post)
The team leader let the team know what was expected of them through direction and command	2.40 ± 0.27	3.10 ± 0.23	0.004
The team leader maintained a global perspective	2.10 ± 0.28	3.20 ± 0.20	0.01
The team communicated effectively	2.7 ± 0.30	3.5 ± 0.22	< 0.001
The team worked together to complete tasks in a timely manner	2.7 ± 0.34	3.6 ± 0.16	0.009
The team acted with composure and control	2.8 ± 0.25	3.4 ± 0.27	0.005
The team morale was positive	3.0 ± 0.30	3.4 ± 0.22	0.002
The team adapted to changing situation	2.2 ± 0.29	3.3 ± 0.21	0.007
The team anticipated potential actions	2.6 ± 0.27	3.2 ± 0.20	0.006
The team prioritized tasks	1.6 ± 0.22	2.4 ± 0.31	< 0.001
The team followed approved standards/guidelines	2.3 ± 0.26	3.2 ± 0.25	< 0.001
Mean total score	2.42 ± 0.34	3.28 ± 0.30	0.001

Overall, the physicians who were regularly trained agreed that they had high confidence in SE management skills and teamwork abilities after training through a combination of curricular learning and in situ simulation training (Table 4).

**Table 4:** Statistics of simulation training evaluation questionnaire [N (%)]

	5	4	3	2	1
Increase interest in emergency medicine	0	25 (41.7%)	20 (33.3%)	15 (25.0%)	0
Learn some operation skills	15 (25.0%)	38 (63.3%)	7 (11.7%)	0	0
Help understand theoretical knowledge	10 (16.7%)	44 (73.3%)	6 (10.0%)	0	0
Improve self-confidence at work	12 (20.0%)	48 (80.0%)	0	0	0
Whether the process is reasonable	0	57 (95.0%)	3 (5.0%)	0	0

## Discussion

To the authors’ knowledge, this study is the first to demonstrate the successful use of curriculum learning combined with in-situ simulation training to teach pediatric SE recognition and management and effective teamwork in emergency regularly trained physicians and residents. This study confirmed a knowledge gap in SE identification and management among ED routinely trained physicians (first year of training) because mastery was not achieved during the initial pre-training assessment. Addressing this knowledge gap is critical as delayed recognition, and appropriate treatment of SE is associated with high mortality and morbidity [21, 31]. A previous study showed that untrained physicians often fail to manage SE adequately [21]. Raoul et al. found that non-compliance with SE treatment guidelines was common in highly standardized SE simulated clinical scenarios.

After completing the coursework combined with the in-situ simulation training, a significant improvement was observed in the performance of all physicians. During the pre-training, most residents were able to ask questions about vital signs, and finger glucose levels, and determine if they needed first-line medications (benzodiazepines), reflecting some of what they learned during medical school and internships skill. Still, 10%-90% of the learners could not complete basic skills in the pre-training, including taking a brief medical history from witnesses, assessing the airway, making sure intravenous fluids were working properly, and seeking help. These issues point to some limitations of traditional medical education and highlight the role of in-situ simulation training.

Previous studies confirmed the value of training approaches that combine curriculum learning with in-situ simulations in SE procedures, communication, and team-based skills. For example, Yara et al. showed that the SBML course significantly improved residents’ SE identification and management skills and that these skills were largely retained and transferred to the hospital setting [18]. In the study by Paulina et al., physicians were found to treat the sequence and frequency of simulated SE according to the systemic airway-breath-circulation-disability-exposure (ABCDE) approach to better protect the airway in a prospective high-fidelity simulation study [32]. In addition, in-situ simulations have been successfully used for communication skills in end-of-life discussions. Robert et al. showed that in situ interprofessional simulation can improve communication and teamwork among professionals in the operating room [33].

In the present study, a combination of applied curriculum learning and in-situ simulation training could improve rescue teams’ leadership, teamwork, and task management skills. In Belgium, a NICU study showed that repeated high-fidelity in-situ simulation training based on curriculum learning had a positive effect on registered nurses’ self-efficacy and self-perceived leadership, and repeated participation in simulation training had a positive effect on these outcomes, regardless of NICU [34]. An in-situ simulation-based study aimed at developing a continuing professional development curriculum in pediatric emergency medicine showed that in situ simulation training had a greater impact on physician learning outcomes and practice [35]. The combination of curriculum learning and in-situ simulation training has many potential advantages over traditional educational methods, including simultaneous satisfaction of team communication, teamwork, direct observation of clinical performance through feedback, and practice of clinical guidelines with minimal patient risk [36, 37].

Finally, this study demonstrated through learner-rated training modalities that combined curriculum learning with in-situ simulations was highly satisfying and that the learners preferred in-situ simulation-based courses over other taught courses, which is consistent with previous research [20, 38]. To the authors’ knowledge, this is the first study to use a resuscitation room to perform an in-situ simulation of SE management in children. Although it was not the study’s main purpose, many team members (nurses and medical assistants) were allowed to improve their practical work skills through a combination of coursework and in situ simulation training.

This study has some limitations. First, the study was conducted in a single institution with few learners, which might reduce the generalizability of the results. Second, the generalizability and portability of the in-situ simulation setup might be limited, as it can only reproduce real-life scenarios to a certain extent, and our results need to be confirmed in real-life clinical settings. Third, our study did not consider the issue of the training cost, which will be taken into account in subsequent studies. Fourth, skill retention was not assessed in the present study. We need to continue to observe the trainees’ maintenance of SE identification and management skills and teamwork ability after the training.

In conclusion, this study showed that training in SE curriculum learning combined with in-situ simulation provides all learners with SE identification and management in children and teamwork skills. Skills learned in the in-situ simulation training can be transferred to the hospital setting. After completing the training, trainees were more confident in SE identification and critical care management.

## Supplementary Information

**Additional file 1:** **Supplementary Table S1.** Education and work experience of Participants. **Supplementary Table S2.** Stages and score of emergency rescue procedures for pediatric SE. **Supplementary Table S3.** The number of times each stage was completed by each group.

## Data Availability

The datasets used and/or analyzed during the current study are available from the corresponding author on reasonable request.

## Abbreviations

SE: Status epilepticus
PICU: Pediatric intensive care unit
ROSC: Cardiopulmonary resuscitation
