# Supplementary material for: Treatment of status epilepticus in pediatrics: curriculum learning combined with in-situ simulations
Source: BMC Med Educ. 2022 Jul 19;22:557. doi: 10.1186/s12909-022-03626-x (PMC9295428; doi:10.1186/s12909-022-03626-x)
Supplement: Supplementary file 1 — Additional file 1: Supplementary Table S1. Education and work experience of Participants. Supplementary Table S2. Stages and score of emergency rescue procedures for pediatric SE. Supplementary Table S3. The number of times each stage was completed by each group. [file 12909_2022_3626_MOESM1_ESM.docx]

**Supplementary Tables**

**Supplementary Table S1.** Education and work experience of Participants

| Participants | | Education and work experience | Number |
| --- | --- | --- | --- |
| Emergency Physician | Regular training Physician | 1st year of postgraduate, 1st year of standardized training of emergency physician | 10 |
|  | Resident | Graduated from postgraduate, emergency work less than 1 year | 10 |
| Emergency Nurse | N1-level nurse | Bachelor's degree, emergency work less than or equal to 1 year, not completed emergency specialist nurse training | 30 |
|  | N2-level nurse | Bachelor's degree, emergency work more than 1 year but less than 3 years, completed emergency specialist nurse training | 10 |

**Supplementary Table S2.** Stages and score of emergency rescue procedures for pediatric SE

| Stage | Content |
| --- | --- |
| Pre-training triage (one triage nurse for pre-test) | 1. ABC (appearance, breathing, circulation) Triangle #1 (21) to assess the patient (completed within 30-40 s, exposed children <3 months of age should be examined); accurately determine the triage level.  2. Simply ask for medical history, including any special contact history, previous treatment, etc., patient in triage grade 1 or 2 is immediately transferred to the emergency room and call rescue physician on duty (skilled use of pager, clear history description).  3. Comfort the family (appropriately). |
| Intensive care  (two rescue nurses: 10-step rescuing critically ill patients) | 1. Conduct correct self-protection according to the situation.  2. Nurse A: straighten the position, suck sputum, give oxygen, pull the guardrail, push the rescue cart, open the vein channel.  3. Nurse B: use the ECG monitor (skilled, first measure pulse oxygen to obtain pulse rate and pulse oxygen value quickly, then connect ECG monitoring lead), and make good records (time to rescue room, heart rate (P), respiration (R), blood oxygen saturation (SpO_2_), blood pressure (BP), medication and time).  Nurse B: 5-min countdown (skilled use of countdown timer), ask patient's family to leave (only one person left), press the "rescue" button, close the door. |
| Emergency room (first 5 min of SE)  (one doctor and two nurses) | First-line doctor:  1. Perform correct self-protection according to the situation;  2. Quick and accurate ABCDE assessment #2.  3. Ask for important medical history.  4. Analyze the condition and give a preliminary judgment.  5. Give oral medical orders, such as sputum suction, oxygen inhalation, the establishment of the venous channel, sedation, and anticonvulsant drugs, and measurement of trace blood glucose (oxygen therapy orders include mode and oxygen flow; sedative and anticonvulsant drugs are first-line drugs and include drug chemical name, dosage, and usage).  6. Communicate with parents or family members, inform them of the condition, and ask redundant family members to leave if necessary.  7. Whether the leader takes full responsibilities.  Rescue nurse:  B: Report the unexecuted part of the doctor's order or repeat the oral order, measure, report, and record the results of microglucose.  A: Repeat the medicine prescribed by the doctor orally, check the name and expiration of the medicine with the doctor or nurse B, report once after preparation and execution, and record the medication and time by oneself or by nurse B after execution. |
| Emergency room (SE for 5 to 15 min)  (one doctor and two nurses) | Nurse B: Report time and ECG monitor data and record.  First-line doctor:  1. According to the respiratory tract status, oral medical orders should be repeated for sputum aspiration, replacement of oxygen inhalation mode if necessary, and re-use of first-line sedative anticonvulsant drugs (oxygen therapy orders include mode and oxygen flow, sedative anticonvulsant drugs include chemical name, dosage, and usage).  2. Communicate with the parents or family members and inform them of the condition.  3. Call the superior doctor, complete the medical record, fill the doctor's order.  Nurse A: repeat the medicine prescribed by the doctor orally, check with nurse B the name of the medicine, expiration period, report once after preparation and execution, and record after execution. |
| Emergency room (SE lasts 15-25 min)  (two doctors and two nurses) | Nurse B: Report time and ECG monitor data and record.  Second-line rescue doctor:  1. Further medical history and physical examination are required to give diagnostic consideration and treatment plan (including sedative anticonvulsant second-line drugs).  2. Instruct nurse B to call for neurologist consultation; whether to take full leader responsibilities.  3. Communicate with parents or family members and inform them of their condition, included in PICU.  Rescue nurse:  B: Call a neurologist for urgent consultation (brief and clear description of medical history and rescue process) and record the time.  A: Open another vein as appropriate; repeat the orally prescribed drugs, check the efficacy period with nurse B, report once after preparation and execution, and record the time after execution. |
| Emergency room (SE lasted 25 - 45 min, apnea, and cardiac apnea)  (three doctors and four nurses) | Nurse B: Report the time and ECG monitor data, record the arrival time of the neurologist consultant.  Second-line rescue doctors:  1. Six-people rescue team (neurologist can participate), assigned with clear tasks;  2. Give oral medical orders for rescue medicines (including chemical name, dosage, and usage);  3. Ask nurse B to consult the anesthesiology department and prepare a tracheal intubation vehicle.  Rescue nurse:  B: Call nurses C and D to participate in the rescue. The timer is changed to a 2-min countdown. Call the anesthesiology department for urgent consultation with concise and clear consultation requests.  A: Repeat the doctor's oral order, check the medicine name and expiration period with nurse C, report once after preparation and execution, and record B after execution. |
| Transfer to PICU after ROSC | Second-line doctor:  1. In-hospital transport preparation instructions (oxygen bags, mini-ECG monitors, resuscitation airbags, or simple ventilators);  2. Communicate with the parents or family members, inform the patient's condition (critically ill), and sign all informed consent forms;  3. Inform the PICU patient of the brief condition, and inform the reception;  First-line doctors:  Replenish the rescue record and doctor's advice.  Rescue nurse  A: Prepare hospital transfer equipment and handover record book for critically ill patients;  B: Notify elevator preparation, record transfer time, and complete nursing record;  C, D: clean and replenish rescue supplies. |

Note 1: In the Pediatric Assessment Triangle (PAT) (21), appearance indicates a child's ability to respond to the environment associated with age and stage of development. For example, the baby's physical examination degree of cooperation, interaction with parents or other caregivers can give comfort, normal eyes or gaze, talk normally, or cry. Circulation to the skin is used to evaluate systemic perfusion. According to fluid loss or blood loss, the color or color type of the child's skin mucosa is determined. In order to ensure the perfusion of vital organs, such as the heart and brain, the compensation mechanism can make the blood perfusion leave the skin and mucous membrane around the body. Therefore, abnormal perfusion of the skin and mucous membrane can be found in the early detection of shock.

Note 2: ABCDE assessment (22). Airway: smooth airway includes smooth breathing (normal pronunciation), partial airway obstruction (wheezing, snoring), complete airway obstruction (breathing hard but no breathing sound). Open airway and clear secretions by forehead and jaw lifting. In case of cardiac arrest, CPR should be performed immediately. If there is a foreign body in the trachea, it should be dealt with it according to the corresponding guidelines. Breathing: adequacy of breathing is assessed by breathing rate, thoracic movement, jugular tension, percussion, skin cyanosis, etc. If there is hypoxia, oxygen is given and assisted breathing and other treatment. If tension pneumothorax is present, a thick needle or pneumothorax puncture is performed immediately in the second intercostal space midclavicular line. Circulation: adequacy of circulation is assessed based on the capillary refill time (CRT), pulse, skin color, perspiration, and level of consciousness. Disability: awareness can be divided into four levels: awareness, audible arousal, pain arousal, and non-arousal, i.e., AVPU, evaluated using the Glasgow Coma Score. Pupil response to light is observed, and blood glucose is determined. Exposure: If there is a change in the level of consciousness, expose the patient's entire skin to any cause, carefully observe the bleeding, wounds, rashes, burns, needle injuries, etc.

**Supplementary Table S3.** The number of times each stage was completed by each group

| Group | Stages | | | | | | |
| --- | --- | --- | --- | --- | --- | --- | --- |
|  | 1 | 2 | 3 | 4 | 5 | 6 | 7 |
| 1 | 10 | 10 | 10 | 9 | 7 | 5 | 5 |
| 2 | 10 | 10 | 10 | 8 | 7 | 6 | 6 |
| 3 | 10 | 10 | 10 | 9 | 7 | 5 | 5 |
| 4 | 10 | 10 | 10 | 8 | 6 | 4 | 4 |
| 5 | 10 | 10 | 10 | 7 | 5 | 4 | 4 |
| 6 | 10 | 10 | 10 | 8 | 7 | 5 | 5 |
| 7 | 10 | 10 | 10 | 8 | 6 | 4 | 4 |
| 8 | 10 | 10 | 10 | 9 | 8 | 6 | 6 |
| 9 | 10 | 10 | 10 | 7 | 6 | 5 | 5 |
| 10 | 10 | 10 | 10 | 9 | 7 | 5 | 5 |
